# Organised Physical Activity in the Forests of the Warsaw and Tricity Agglomerations, Poland

**DOI:** 10.3390/ijerph16203961

**Published:** 2019-10-17

**Authors:** Emilia Janeczko, Roman Wójcik, Wojciech Kędziora, Krzysztof Janeczko, Małgorzata Woźnicka

**Affiliations:** 1 Department of Forest Utilization, Institute of Forest Sciences, University of Life Sciences in Warsaw, Nowoursynowska 159, 02-776 Warsaw, Poland; mwoznicka@wl.sggw.pl; 2 Department of Forest Management Planning and Forest Economics, Institute of Forest Sciences, University of Life Sciences in Warsaw, Nowoursynowska 159, 02-776 Warsaw, Poland; roman.wojcik@wl.sggw.pl (R.W.); wojciech.kedziora@wl.sggw.pl (W.K.); krzysj@wl.sggw.pl (K.J.)

**Keywords:** urban forests, physical activities, health benefit, organised sport

## Abstract

*Research Highlights:* The achieved results indicate that in the period of 2010–2016, the number of sporting events (running, cycling and walking) as well as the number of participants has increased many times. *Methods:* A geostatistical method, i.e., kriging, was used to check the distance-based relationship between agglomerations proximity and sporting events. The questionnaire surveys were used to determine the reasons for physical activity in the forest. *Results:* The analysis of the impact of the scope of the urban agglomerations on the number of organised physical activities proved that, in both cases (Warsaw and Tricity), most events take place in forests located close to these cities. The larger the distance to Warsaw or Tricity, the lower the number of sporting events and the lower the number of participants. The reasons why people take up physical activity are mainly to improve their health and physical condition, reduce stress and improve wellbeing. *Conclusions:* The conducted research indicates a significant increase in the importance of non-productive functions of forests located in the vicinity of urban agglomerations, as well as the need for continuous monitoring of actions taken by foresters to promote active lifestyles.

## 1. Introduction

Urbanisation and insufficient physical activity are leading to increased physical and mental health problems in many cases and in many societies. These problems lead to lower labour productivity and increased absenteeism and they contribute to increased expenditures for health care systems. Opportunities to reverse these trends can be seen in the development of green infrastructure and the promotion of active lifestyles. The World Health Organization [1] states that one third of adults in the world do not attain the recommended levels of physical activity. Moreover, it is estimated that in Europe more than one third of adults have an insufficient level of physical activity. Around 23.1 million deaths each year (ca. 10% of all deaths each year) and 8.3 million disabilities are caused by a lack of physical activity [1]. Ortega et al. [2] suggested that physical education in early life-stage plays an important role in promoting physical activity and healthy lifestyle behaviours throughout adulthood. Regular physical activity every week reduces the risk of diabetes, coronary heart disease, hypertension, depression, and breast and colon cancer, and increases life expectancy [3], ensures mental wellbeing, and helps to prevent osteoporosis and to maintain viability in older people [4]. Furthermore, a large number of studies [5,6,7] have proved that physical activity in a natural environment might bring greater health benefits than physical activity in other places. Son and Ha [8] implied that expanding contact with nature provides the benefits of better social and emotional capabilities in modern society. Tyrväinen [9] points out that while the health benefits of healthy adults’ contact with nature are well documented, there is little research into the impact of nature on recovery from sicknesses. Research in this direction was undertaken by Bielinis and Jaroszewska [10]), Taylor et al. [11], Kuo and Taylor [12].

Nature has a regenerating effect on many people, reducing stress levels [13], and outdoor activities in urban green spaces might thus be a promising means to lower stress levels [14]. Research by Korpela [15] and Tyrväinen et al. [16] proved that natural areas raise positive feelings among people. Korpela [17] also demonstrated that the process of regeneration amid natural scenery, and especially in forests, is much longer lasting than the regeneration offered by city parks or other open recreational areas in cities. Pirnat and Hladnik [18] suggested that only forests that are close to nature and which have relatively low silvicultural inputs offer ecosystem services that are sufficient to fulfil the supply and demand of health benefits of the expanding urban population. The relationship between the natural environment and human well-being and health is the subject of interest of many organisations operating in the public health and environmental sectors [6]. Recently, there has been considerable and increasing global attention towards using forest environments as places for recreation and health promotion [19]. In Poland, as in other European countries in which forests traditionally serve as a source of timber, forests are increasingly functioning as recreational areas. Poland is dominated by open, public forests, which are easily accessible due to the area they occupy (about 30% of the territory of Poland). Increasingly, they are places for various forms of physical activity, such as walking, trekking, cycling, or running, that are undertaken as organized activities which can be defined in a subset of physical activity which is structured, goal-oriented, competitive, and contest-based [20]. Research on people’s preferences for active recreation and exercise [21,22,23,24,25,26] undertaken in Poland and other countries shows that physical exercise, as well as participation in organised sports, is far more popular among urban inhabitants than among inhabitants of rural areas. Roemmich et al. [26] determined that less physical activity among rural residents may contribute to rural–urban health disparities. The phenomenon of mass public participation of Poles in various organised physical activities in forests is probably correlated with the proximity of large cities. We expect that the demand for forest use for the development of organised physical activity will change with increasing distance from large cities.

The aim of this study is to determine the spatial extent of organised physical activities in the forests adjacent to large cities, taking the large Polish urban agglomerations of Warsaw and Tricity as examples. The following research questions were formulated:

What is the scale (number of events and number of participants) of organised physical activities in the forests surrounding these large cities?

Does the distance from the city affect the intensity of organised physical activities in the forests? What are the reasons for the interest of the inhabitants of large cities in organised physical activity in forests? 

Answers to these questions will be provided by using data on the number of sporting events in the forests and the number of participants gathered for the needs of these studies and using appropriate GIS tools and surveys conducted among persons interested in the use of the forest for sports and active recreation purposes.

## 2. Methods 

**Figure 1:**
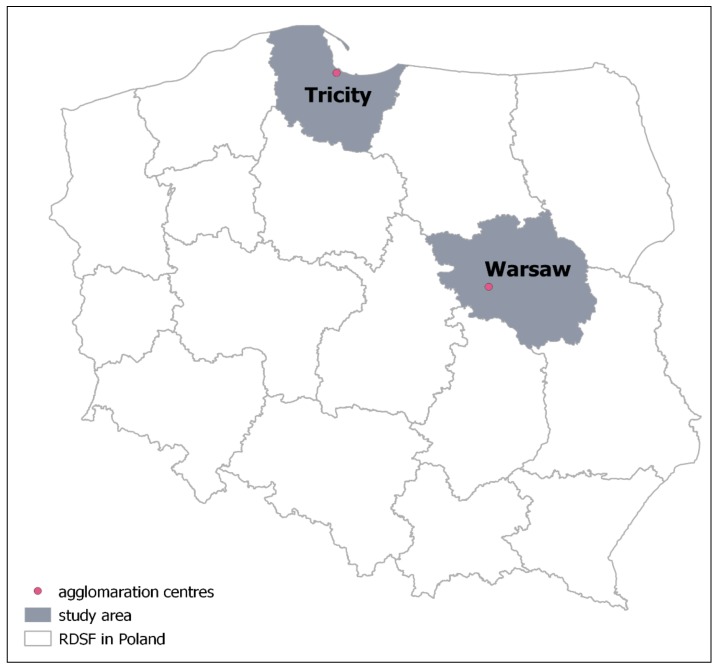
Location of Warsaw and Tricity on the background of the organisational divisions of state forests in Poland.

In Poland, in accordance with the act on spatial planning and development of 2003 [27], a metropolitan area is the area of a city and its direct surroundings, functionally connected, set up in the concept of national spatial planning [28]. The metropolitan area of Warsaw occupies an area of 6206 km^2^, 8.3% of which is occupied by the city of Warsaw. In 2015, the population of the Warsaw metropolitan area reached 3,126,341 people, which accounted for 58.4% of the population of the Masovian Province [29]. The Warsaw urban agglomeration is monocentric and is located in mid-Eastern Poland, as shown in Figure 1. The forests in the territory of the Warsaw urban agglomeration are supervised by the Regional Directorate of State Forests (RDSF) in Warsaw. The RDSF in Warsaw is divided into 14 forest districts. The total area of the state-owned forests administered by the RDSF in Warsaw is slightly larger than 183,000 hectares, while forests of other ownership make up an area of almost 221,000 hectares. The total forest cover of area administrated by RDSF in Warsaw is 24.7%. Forest resources (percentages) in each forest district are not similar. Larger forest complexes are located in the northern and eastern part of this area. The dominant tree species is pine (80.3%). The percentage of oak (7.4%), papillary birch (5.6%) and alder (5.4%) is significant here. Stands at the age of 60–80 years dominate. About 60% of the forest area is covered by stands on coniferous and mixed coniferous forest habitats and about 40% on broadleaved and mixed broadleaved forest habitats.

As also shown in Figure 1, the Tricity urban agglomeration lies in Northern Poland, on Gdańsk Bay. Its central part is comprised of Tricity, i.e., the polycentric area of the cities of Gdańsk, Sopot, and Gdynia. The Tricity Metropolitan Area covers 3071 km^2^, and is inhabited by 1,224,787 people [30]. Forests of the Gdańsk urban agglomeration are administered by the RDSF in Gdańsk, and are comprised of 15 forest districts. The area administered by the RDSF in Gdańsk is 304,000 hectares, the forest area is 284,700 hectares, and the forest cover amounts to 30%. This forest cover is also not evenly distributed as in the previous case. The highest forest cover is found in the northern and south-western part of the Directorate. The dominant species is also pine (69.0%), although its share here is 11.3% smaller than in the range of RDSF Warsaw. In the forests around the Tri-City, the percentage of beech (13.9%), which has its natural habitat in this area, is significant. The forests around the Tricity are more age-differentiated than those near Warsaw. The proportion of stands over 100 years old (22.2%) is higher than in the forests around Warsaw (9.2%). In this area, there are about 60% of broadleaved forest habitats and 40% of coniferous forest habitats.

Empirical data on sporting events organised in forests in the years 2010–2016 (the number of events organised into the categories of running, cycling, and walking events, and the number of participants for each event) were provided by all forest districts of the RDSFs in Warsaw and Gdańsk. For this purpose, a detailed analysis of applications and applications for permission to organize a sports event in the forest submitted by external entities (sports organizers: e.g., event and marketing agencies, local authorities, local action groups) to the forest inspectorate was carried out. Such documents each time contained information on the type of project, date of the event, estimated attendance of participants, etc. 

An analysis of the influence of the two large urban centres (Warsaw and Tricity) on the spatial range of the organised physical activity in forests was performed on the basis of geostatistical methods, which allowed the principle of allocation of the analysed feature (the number of events and the number of participants) to be recognised in space. Geostatistical methods allow to the analysis of spatial correlation of distributed data [31]. It was assumed that the points with the attached values concerning the events and numbers of participants were the geometrical centres of the forest districts. The first step in the empirical analysis was to geocode location and event data (number of events and number of participants in each event). Geostatistical analyses were performed with the use of the ArcGIS v. 10.0 software (Esri, Redlands, CA, USA). Next, distance zones were calculated around each of the two urban agglomerations at a radius of 100 km [32]. The method of universal kriging was applied enabled information on the spatial allocation both for the analysis of the number of events and the analysis of the number of participants. Then, a distribution map of the examined feature was interpolated for the entire observed area. Identifying the reasons why people participate in organised physical activities in forests required a diagnostic survey method. The survey was conducted in the period from 1 October to 20 November 2016 by means of an online survey. The research was conducted with the use of financial resources for the statutory activity of the Faculty of Forestry at the University of Life Sciences in Warsaw. The questionnaire was available at https://www.webankieta.pl/. The survey involved 346 persons over 18 years of age. The questionnaire was distributed via social networking sites (e.g., Facebook) among people living within the range of both agglomerations, interested in sports and active recreation in forests. The invitation to participate in the survey was addressed to thematic groups of running persons. The choice of this group of forest users was connected with the fact that running is currently the most popular sport in Poland, as shown by social research conducted in 2013 [33]. However, it should be noted that the surveyed group of respondents is not representative of all Poles or Polish Internet users.

The questionnaire consisted of metric questions used to determine the structure of respondents and fifteen basic questions concerning: the advantages and disadvantages of running in forests, the frequency of running in forests, the preferred season, the role of the State Forest in promoting sports activities, and so on. The following question with the possibility of selecting up to three answers was important for the realisation of the aim of this study: 

“Please indicate three reasons why you take up physical activity in the forest / improvement of health, physical condition / stress reduction / improvement of wellbeing/ physical activity allows to lose unnecessary calories / improvement of sporting performance, competition/ feeling close to nature/ a sense of escape, freedom/ physical activity raises its own value/ a sense of fun/enjoyment/ other reasons.”

All answers were anonymous and confidential. Testing of the respondent’s opinion conformity with regard to their gender, age and place of residence was performed with the chi- square homogeneity test in the Statistica v.13 package (Dell Inc. 2016, TX, USA). The verification of statistical hypotheses was carried out at the materiality level of 0.05. The strength of dependence between two qualitative variables was assessed using the quota coefficient (C).

## 3. Results

In forests adjacent to Warsaw, in the years 2010–2016, a total of 537 sporting events took place, with 127,579 participants. In the same period, in the surroundings of Tricity, a total of 603 events were organised, with 124,410 participants. It was observed that the number of sporting events in the forests of the Warsaw RDSF rose year-on-year, from 34 events in 2010 up to 112 in 2016 (Table 1). The number of participants rose simultaneously, from 7730 in 2010 to 27,817 in 2016. The situation in the forests of the Gdańsk RDSF was similar. The number of events in the analysed period rose from 14 in 2010 up to 172 in 2016. The number of participants of sporting events also grew year-on-year, from 2340 in 2010 to 33,509 in 2016. 

Of the sporting events organised in forests in proximity to Warsaw in the study period, running competitions dominated (250 events), making up 46.5% of all sporting events in this area. These attracted a total of 51,347 participants, equal to 40.2% of all participants of sporting events in this area in the study period. Cycling events (227 events) made up 42.3% of all sporting events organised in this area, attracting 69,462 participants (54.4% of all participants). Walking events (60 events) represented 11.2% of all sporting events in the area, attracting a total of 6770 people (5.3% of participants). A similar pattern of sporting events was observed in forests encompassing the Tricity urban agglomeration in the study period. Running events also dominated here (335 events), accounting for 55.6% of all sporting events organised in this area. The total number of participants in running events in the area was 64,578, that is, 51.9% of all participants of sporting events in the area in the study period. A total of 144 cycling events took place in the study period (23.9% of all events in the area), with a total of 21,787 participants (30.6% of all sporting event participants in the area). A total of 124 walking events (20.6%) were held, with a total of 38,045 participants (17.5%). 

**Figure 2:**
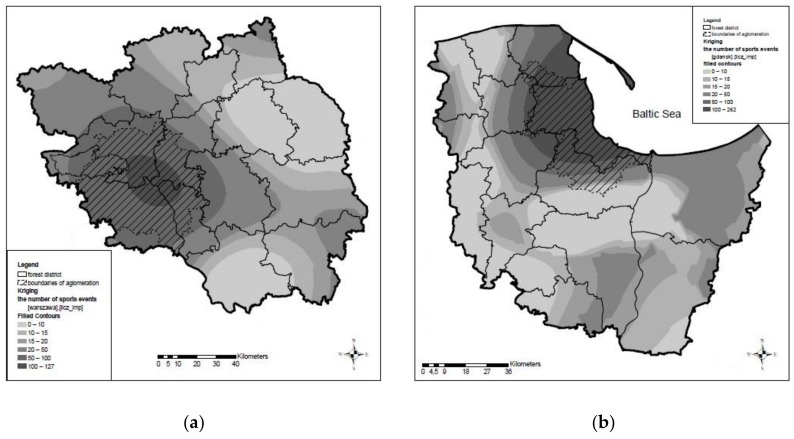
The spatial extent of organised forms of physical activity in forests of the urban agglomerations of (**a**) Warsaw and (**b**) Tricity.

By analysing the range of influence of the huge urban agglomerations of Warsaw and Tricity on the number of sporting events organised in forests, it was shown that most events take place in forests directly surrounding these cities. The greater the distance to Warsaw or Tricity, the lower the number of sporting events and the lower the number of participants. The greatest number of events takes place in the region up to 50 km from the centres of both urban agglomerations. The majority of the area of the Warsaw urban agglomeration, that is, 5522 km^2^ (89% of the total area), lies within the scope of the second level of intensity of sporting events (51–100 events), see Figure 2a. The area of the Warsaw urban agglomeration with the first level of intensity (more than 100 events) is 579 km^2^ (9.3% of the total area). The remaining 105 km^2^ (1.7%) falls into the third intensity level (21–50 events). The majority of the area of the Tricity urban agglomeration, that is, 2662 km^2^ (86.7%), belongs to the first level of intensity of sporting events, see Figure 2b. Around 250 km^2^ (8.1%) of the Tricity urban agglomeration belongs to the second level of event intensity, and 85 km^2^ (2.8%) to the third level. The remaining part of the Tricity urban agglomeration, that is, 74 km^2^ (2.4%), is within the zone of 0–20 sporting events (0–10 events: 1.2%; 11–15 events: 0.5%; 16–20 events: 0.7%). 

**Figure 3:**
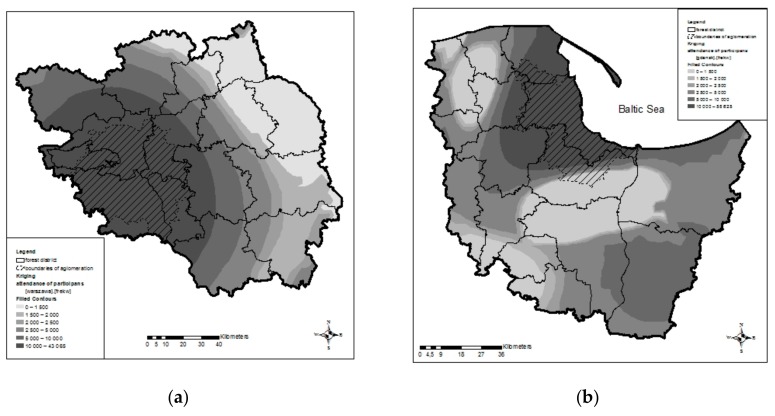
The urban agglomerations of Warsaw (**a**) and Tricity (**b**) and participation at organised forms of physical activity in forests.

**Figure 4:**
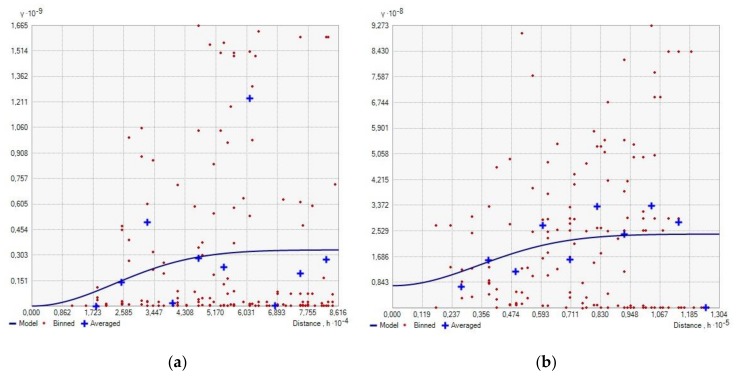
Semivariogram illustrating the relationship between the number of participants and the distance from urban agglomerations of Warsaw (**a**) and Tricity (**b**).

Considering the number of participants of organised forms of physical activity in forests, it can be seen that the participation rate at sporting events was more than 10,000 during 2010–2016 in the entire area of the Warsaw urban agglomeration, see Figure 3a. Similarly, the major part of the Tricity urban agglomeration area, that is, 2882 km^2^ (93.8%), is also located in the zone where the number of participants at sporting events exceeded 10,000 in the same period. An area of ca. 75 km^2^ (2.4%) is in the zone with 5001–10,000 participants. The remaining area of 114 km^2^ (3.7%) lies in the zone of ≤5000 participants, see Figure 3b. The impact of the huge urban agglomerations on the number of sporting events and the number of participants is visible not only on maps (Figure 2 and Figure 3), but also on semivariograms, which confirm that the longer the distance to Warsaw (Figure 4a) or Tricity (Figure 4b), the lower the participation at the previously described organised events. 

The conducted surveys show that the organized physical activities in forests are attended mainly by city dwellers (76.3%), including 42.8% of respondents coming from cities with more than 500 thousand inhabitants, 23.7%—from cities with up to 100 thousand inhabitants, and 9.8%—from cities with between 100 thousand and 500 thousand inhabitants. Rural areas were inhabited by 23.7% of respondents. They are more often men, accounting for 67.6% of survey participants, see Table 2. The largest group of respondents (44.8%) were people aged 35–44, the smallest (4.8%)—aged 18–24. The share of respondents aged 25–34 amounted to 32.7%, of respondents aged 45–55 to 11.6%, and of respondents aged over 55 to 6.1%. The most frequently mentioned reason why people took part in organized physical activities in forests was the improvement of health and physical condition (85.5%), followed by stress reduction (70.8%), improvement of wellbeing (46%) and the need to lose weight (36.4%). Other factors such as e.g., “improvement of sports results”, “sense of closeness to nature” or “sense of freedom” were indicated by 28.3%, 18.2% and 7.8% of respondents respectively. Other factors, such as “physical activity raises its own value”, “is important for pleasure”, while “other reasons” were indicated by 4.3%, 1.4% and 1.2% of respondents respectively. The statistical analysis shows that the respondents’ views on the reasons why they participate in organised physical activity are differentiated according to gender (C = 0.145, *p* = 0.006) and place of residence of the respondents (C = 0.153, *p* = 0.039). Age, as well as seniority in running did not differentiate the opinions of respondents in statistical significance. Women at a similar level as men pointed out that among the important reasons for undertaking physical activity in forests were: “improvement of health and physical condition” and “improvement of wellbeing”. A much higher percentage of women (75.9%) than men (68.4%) indicated “stress reduction” as an important factor of taking up physical activity in the forest. Women, to a greater extent than men, pointed to reasons such as losing weight, self-esteem or being close to nature. The opinions of women and men on factors such as “improvement of sport results, competition” were clearly different. In this case, the percentage of men (32.9%) was much higher than that of women (18.8%). Taking into account the place of residence, it can be concluded that the views of respondents differ substantially, as in the case of gender, with regard to the willingness to improve their sporting results. This factor was indicated by 35.8% of respondents from cities with more than 500,000 inhabitants, 29.4% from cities with 100,000 to 500,000 inhabitants and 23.2% from cities with up to 100,000 inhabitants and only 19.5% from rural areas. “The improvement of health and physical condition” was more often indicated by the inhabitants of rural areas than by the inhabitants of cities. On the other hand, “stress reduction” was most often indicated by city dwellers with over 500,000 inhabitants. “Improving of wellbeing” was indicated at a similar level by both rural and large cities with over to 500,000 inhabitants. 

## 4. Discussion

In recent years, the patterns of spending free time in Poland have changed significantly. More and more people prefer a healthy lifestyle. The expression of this is, among others, the increase in the value of the sports market. The turnover of the sports articles sector already amounts to over PLN 8 billion. At the same time, an increase in the value of the sports sponsoring market is also observed. In 2017, there were 870.7 million PLN. This is 4.6% more than in 2016 [34]. New trends result from the development and greater accessibility of recreational infrastructure, organization of large sports events and more affordable offer of sports equipment [35]. A very important factor in forming collective and individual habits related to the society’s physical activity is adequate policy, resulting in legislatory or regulative actions undertaken by governmental or non-governmental organisations [36]. Keeping in mind this background, the role model can be the activity of the State Forests National Forest Holding. This institution administers 80% of the forest area in Poland, and plays a significant role in the development of physical activity, including recreational and sporting activity, on forest grounds. The State Forests in cooperation with various running clubs realize actions aimed at promoting running, healthy lifestyle and encouraging to practice sports in forest areas (e.g., “BiegamBoLubięLasy”, literally translated—“I run because I like forests”,). Sporting events organised or co-organised in forest areas by this institution firstly constitute a way of making forests accessible to a large group of people, and secondly are a major marketing element, due to which the cooperation of foresters with various social partners is being developed. Previous research has proved that forests are eagerly used for various types of physical activity. Environmentally friendly exercises such as jogging, walking, and cycling in green spaces offer people many benefits in terms of wellbeing, and are also favourable to social interaction, with the potential to promote a general sense of community, decrease feelings of loneliness, and increase social support [37,38]. Increased public awareness of the impact of physical activity on the quality of life, health and psychological well-being of the individual enhances the development of active recreation and sporting activities such as running [39]. Research indicates that urban green areas are recognised as suitable settings for running and jogging [40]. In the forests of the urban agglomerations of both Warsaw and Tricity, the number of running events dominated those of cycling or walking. However, this dominance was not as clear in the areas surrounding Warsaw; compared with the forests surrounding Tricity, in those around Warsaw, cycling was also a very popular activity. A study of recreational preferences conducted in forests neighbouring the city of Warsaw [41] showed that interest in cycling for recreation has been high for a longer period. Recently, it increased profoundly. This might be the reason why the participation at cycling events is much higher (over 10,000 people between 2010–2016) than at running events. The situation in forests surrounding Tricity differs little from that in forests surrounding Warsaw. The numbers of walking and cycling events in both of these places are similar, in both cases being much lower than the numbers of running events. Even by adding the numbers of participants of walking and cycling events, the sum does not reach the number of participants of running events. 

The survey results show that the percentage of men participating in organized physical activities in forests is higher than that of women. The obtained gender proportions are also reflected in other, similar studies. For example, Dzięgiel [33] found that in the Polish running community women constitute about 20–30%. Also, in the Dusiński [21] and Report—National census of runners [42] studies, the share of men was higher. Among forest runners there are mainly middle-aged (35–44 years) or younger (25–34 years) people. Similar results were obtained by Dusiński [21]. Data from mass runners’ ranges also point to the predominance of runners’ ranges from 30 to 40 years of age [22]. According to the Report—National Census of Runners [42] the largest group of runners were those aged 25–34 and 35–44. As the research has shown, runners are mainly city dwellers. Dusiński [21] made similar observations. Dzięgiel and Tomanek [22] also found that 81% of respondents taking part in mountain running events came from cities, including 45% from cities with more than 200,000 inhabitants. The National Census of Runners 2014 [42] also confirms that runners are mainly (84%) city dwellers. 

Motives for undertaking specific sports disciplines, as well as forms of sports tourism (sport tourism) were dealt with, inter alia, by Shipway and Joner [43], Gibson [44], Weed [45], Wann at all [46], Getz and McConnell [47]. The Akpinara study [48] showed that green area (especially greenways) ware mainly used for recreational, to do sport, health (i.e., to keep healthy, to reduce stress and relax) and leisure activities (to enjoy the weather and get fresh air). The study by O’Brien and Forster [49] shows that the main motivations for people to get involved in activities were to be physically active in nature (85%), for enjoyment (77%), to get fit (62%) and to improve health (61%). The qualitative research identified further motivations of undertaking a social activity, people wanting to challenge themselves, and having a choice of activities. Our research indicates also that the most frequently reasons why people take physical activity in forests are: improvement of health and physical condition, stress reduction and improvement of wellbeing. Motives for engaging in physical activity in forests may be different due to the personal characteristics of respondents. Our research shows that gender and place of residence are the most important in this context. It was interesting to note that women, to a much greater extent than men, pointed to stress reduction as an important reason for undertaking physical activity in the forest. Numerous studies have shown that women and men differ in their perceptions nature [50,51,52,53]. The study Hedblom et al. [54] points to physiological differences in gender related to stress reduction in urban green areas. Many researchers noted [55,56] that males tend to score higher on measures of competitiveness than do females. Therefore, improvement of sporting performance, competition, also in running, is more often the domain of men than women, and therefore their participation in various types of occupations is higher. This probably explains the observed differences in the respondents’ views on the reasons for their interest in participating in organized sports events in the forests. Competition in sports events and the willingness to improve their own sports results is, as the research has shown, much stronger in the group of city dwellers, especially those living in large cities with a population of more than 500 thousand people. Generally, for both rural and urban residents, the most important reasons for physical activity in forests were “the improvement of health and physical condition”, “reduction of stress" and „improvement of wellbeing”. It is interesting that the first of these factors was more often pointed out by rural inhabitants. As it results from the Strategy [57], one of the development problems of rural areas in Poland is the insufficient quality of public services. It is lacking here: public infrastructure adapted to the needs of people with disabilities, health service providers are too small and insufficiently equipped. There is little possibility of building sports facilities with the own funds of municipalities. These specific features of space mean that the inhabitants of rural areas perceive contact with nature and physical activity in the forest as an opportunity to maintain/improve their health and physical condition. On the other hand, the number of stress factors in cities is probably higher than in rural areas. Hence, "stress reduction" was more often indicated by inhabitants of large cities. We did not notice any differences resulting from the age of respondents. Perhaps it was related to the low number of the youngest respondents aged 18–24 (4.8%) and persons aged over 55 (6.1%). 

A number of studies have analysed the relationship between physical activity and spatial features, i.e., the presence of proper infrastructure such as walking paths and sports facilities [58,59,60], or the distance from the dwelling place [7,61]. There are many factors influencing the attractiveness of forests as a place of organised physical activity. Explanation of all these aspects is important for understanding the scope of forest ecosystem services and planning forest functions. Our research focused mainly on the impact of a large city on the scale of organized physical activities in the forest. We have established that the closer to the city, the higher the number of organized physical activities and the higher the attendance of participants. As noticed in [62], not much is known about the relationship between the size of a city and the level of physical activity of its inhabitants, and the number of studies conducted on this topic is still insufficient. Our present research indicates an increasingly important role of forests in creating the sporting opportunities of a city for certain activities such as running, cycling, and walking, which implies interesting ideas for the sporting policy and, moreover, spatial policy, of cities. Cities invest in sport objects for various reasons, such as ecological development, tourism, societal development, the enhancement of the city’s image, and the improvement of quality of life. 

The needs and expectations of cities regarding sport development may differ, considering that each city has its own unique features [63]. The accessibility of open spaces such as forests, both in cities and in suburban areas, along historical determinants, location, weather, transport infrastructure, etc., might have a crucial meaning in the planning of urban development based on sport objects and the fostering of physical activity among urban inhabitants. 

## 5. Conclusions

Forests in proximity to huge urban agglomerations are commonly used as recreation and sports areas. In Poland, among all types of sports and sport competitions realised in forests, the most popular are mainly running, cycling, and walking events. As ascertained in the course of research in the years 2010–2016, an observed three-fold rise in the number of sporting events in forests surrounding Warsaw, and a 10-fold rise in the number of events in the area around the Gdańsk RDSF, along with a simultaneous rise in the number of participants (over three-fold in Warsaw and 14-fold in the proximity of Tricity), explicitly indicate the growing importance of the social functions of forests in the vicinity of urban agglomerations. The high public interest in and benefits from organised physical activity in forests suggests that forests within urban agglomerations should be integrated into public health systems and policies that promote the mental and physical health of city dwellers. 

The conducted research and the achieved results allow the following conclusions: 

(1) Forests neighbouring large cities are under immense recreational pressure and serve more often as places to realise various sporting and recreational activities of a competitive character, mainly related to running and cycling.

(2) The closer a forest is to the city borders of places like Warsaw or Tricity, the larger the number of sporting events and the greater the number of participants. In forests located peripherally to urban agglomerations, there are much fewer sporting events and participants. 

(3) Forest users are highly aware of the benefits of physical activity in forests, such as: improvement of health and physical condition, reduction of stress or improvement of wellbeing.

(4) Social views on the reasons for interest in participation in organized physical activities in the forest are determined by the gender and place of residence of respondents. Gender differentiated the opinions of respondents on “stress reduction” and “improvement of sports results and competition”. The biggest difference in the opinions of rural residents and large cities with more than 500,000 inhabitants was in the “improvement of sporting results and competition”.

(5) The conducted research and achieved results can be the factor for delimiting forest ecosystem services and for determining the intensity level of their development in the scope of city impact. 

## Figures and Tables

**Table 1:** The number of sporting events and the number of participants in the vicinity of the urban agglomerations of Warsaw and Tricity in 2010–2016.

Year	Forests in Proximity to Warsaw				Forests in Proximity to Tricity			
	No. of Events	%	No. of Participants	%	No. of Events	%	No. of Participants	%
2016	112	20.9	27,817	21.8	172	28.5	33,509	26.9
2015	105	19.6	22,877	17.9	121	21.9	30,619	26.1
2014	102	19.0	25,712	20.2	111	20.2	24,454	20.5
2013	78	14.5	20,092	15.7	87	15.1	17,438	14.4
2012	62	11.5	14,130	11.1	30	6.5	5350	5.9
2011	44	8.2	9221	7.2	25	5.1	4760	4.2
2010	34	6.3	7730	6.1	14	2.7	2340	1.9
Total	537	100.0	127,579	100.0	603	100.0	124,410	100.0

**Table 2:** Views of respondents on factors determining their physical activity in forests depending on gender and place of residence.

Please Indicate These Reasons Why You Take Up Physical Activity In The Forest	Together (%)	Sex		Place of Residence			
		F	M	Village	A City with up to 100,000 Inhabitants	A City of between 100,000 and 500,000 Inhabitants	City over to 500,000 Inhabitants
Improvement of health and physical condition	85.5	85.7	85.5	89.0	85.4	82.4	84.5
stress reduction	70.8	75.9	68.4	70.7	69.5	67.6	72.3
improvement of wellbeing	46.0	44.6	46.6	43.9	46.3	58.8	43.9
physical activity allows to lose unnecessary calories	36.4	38.4	35.5	41.5	42.7	29.4	31.8
improvement of sport results, competition	28.3	18.8	32.9	19.5	23.2	29.4	35.8
feeling close to nature	18.2	20.5	17.1	20.7	17.1	26.5	15.5
a sense of escape/freedom	7.8	7.1	8.1	4.9	12.2	2.9	8.1
physical activity raises its own value	4.3	6.3	3.4	4.9	2.4	0.0	6.1
a sense of fun/enjoyment	1.4	0.9	1.7	2.4	0.0	0.0	2.0
other reasons	1.2	1.8	0.9	2.4	1.2	2.9	0.0
